# Exploring the Motivation Behind Discrimination and Stigmatization Related to COVID-19: A Social Psychological Discussion Based on the Main Theoretical Explanations

**DOI:** 10.3389/fpsyg.2020.569528

**Published:** 2020-11-13

**Authors:** H. Andaç Demirtaş-Madran

**Affiliations:** Faculty of Communication, Başkent University, Ankara, Turkey

**Keywords:** discrimination, stigma, pandemic, COVID-19, social identity, health psychology, infectious disease, social psychology

## Abstract

The novel coronavirus (COVID-19), was first detected in Wuhan province in China during late December 2019 and was designated as being highly infectious. The World Health Organization (WHO) labeled it a “pandemic” on March 11, 2020. Throughout human history, experience has shown that prejudices and viruses spread simultaneously during a viral pandemic. Outgroup members have been associated with various diseases and non-human vectors of diseases. Some epidemics have been named according to various outgroups, just as the novel coronavirus has been referred to by some as the “Wuhan virus” or the “Chinese virus.” Associating a virus with a sociodemographic group builds a false illusionary correlation, which can lead to stigmatization and discrimination. Pandemics can also stimulate violent xenophobic reactions. Besides the obvious harmful consequences for the individuals targeted, pandemic-related discrimination also affects the spread of the virus through its effect on public attitudes toward prevention and restriction, health service procurement, and in the establishment of health-related policies. It is important to first understand the relevant concepts and processes, and also to understand the underlying causes of discrimination in order to fight it. Social psychology offers multidimensional and comprehensive explanations of prejudice and discrimination. This review’s primary aim was to examine the motivations behind COVID-19-related discrimination based on social psychological perspectives. In line with this aim, the review first defines discrimination in detail, plus the related concepts and main social psychological theories on prejudice and discrimination. Then, pandemic-related discrimination in light of past experiences is discussed and explanations put forward for the theoretical perspectives and inferences specific to COVID-19. Finally, recommendations are made in order to prevent and combat discrimination related to infectious diseases.

## Introduction

Despite notable innovation in modern medicine to eradicate pandemic diseases, infectious diseases are still one of the main causes of death and remain an ever-present threat to global humanity (Bloom and Cadarette, 2019). The novel coronavirus, named as COVID-19 (Coronavirus Disease, 2019), was first detected in late December 2019 in the Wuhan province of China. It is caused by a zoonotic beta-coronavirus (SARS-CoV) and is described as being highly infectious (Zhong et al., 2020). It is also viewed as a relative of both Severe Acute Respiratory Syndrome (SARS) and Middle East Respiratory Syndrome (MERS; Sohrabi et al., 2020). The COVID-19 outbreak was announced as a Public Health Emergency of International Concern on January 30, 2020 (World Health Organization, 2020a), and the WHO (World Health Organization, 2020b) labeled it as a “pandemic” on March 11, 2020. The pandemic rapidly spread worldwide, with the virus having reached 216 countries and territories as of September 15, 2020, with a total of 29,155,581 confirmed cases and 926,544 deaths attributed to the disease, according to the WHO (World Health Organization, 2020c).

There are many points where COVID-19 has differed from other pandemics, and which have resulted in increased negative effects in many areas. When compared to previous diseases such as SARS, MERSs, and even Ebola, the novel coronavirus has a lower mortality rate; however, the infection spreads far more easily and is therefore much more pervasive. The death rate from COVID-19 exceeded five times that of SARS after just 3 months (Callaway et al., 2020). While measures applied during almost all previous pandemics were mostly limited to rules for personal hygiene and sanitation, in the novel pandemic, localized, regional, and even national lockdowns, physical (social) distancing rules, travel restrictions, and other measures were applied almost globally; although the measures varied significantly both regionally and at the country level. One significant consequence could be considered as prolonged interruptions to face-to-face education, affecting national and private institutions from kindergarten right through to universities. Such widespread measures result in far greater effects on mental health, intergroup and international relations, education, as well as the global economy. These long-term lockdowns have been seen to exacerbate the differences afforded by privilege and wealth, as those without secure housing, clean drinkable water, sanitation, and reliable employment face increased vulnerability during the social/health-related measures introduced in many countries during such a pandemic. Those living in impoverished conditions often lack access to appropriate medical and/or cleansing products, and also face inabilities to meet social distancing or bubble/quarantine living requirements due to shared, insecure, overcrowded accommodation, or even living without any formal accommodation in unsanitary conditions. Groups such as health workers and medics have been unable, due to their professional responsibilities, to maintain the prescribed social distance from others, and have been exposed to significant levels of discrimination, even though they work under very difficult conditions for the well-being of the public at large, while facing increased personal risk. Although they have been widely praised as heroes, they have also been stigmatized, avoided, and excluded due to their being perceived as sources of infection (Taylor et al., 2020; World Health Organization, 2020d), which was also similarly observed during previous outbreaks such as SARS (Bai et al., 2004).

The epidemic has caused not only significant death and serious health issues, but also severe economic, educational, psychological, and social impacts, and even international crises. Undoubtedly, one of the most “permanent” and “resistant” issues seen during this pandemic is discrimination.

The novel coronavirus has also begun to be referred to by some as the “Wuhan virus” or the “Chinese virus.” This practice, which has become habitual throughout history, is known to cause discrimination and stigmatization. The WHO offered guidelines in order to combat this practice, emphasizing that viruses can infect all human life regardless of their location. Nevertheless, certain political figures worldwide have regularly associated COVID-19 with China, and individuals of Asian descent have been subjected to racist attacks (*Nature*, 2020). Ethnic outgroups are often accused of causing or helping spread pandemics, and these acts can ignite underlying xenophobic tendencies (Oldstone, 1998).

COVID-19 has significantly impacted Black, Asian, and minority ethnic and migrant groups more than other population groups (Devakumar et al., 2020). During the initial spread of the pandemic, numerous instances of “Sinophobia” were reported worldwide. Also it has been an increase in homophobia, Islamophobia, and antisemitism. With the novel coronavirus spreading on a global scale, racism, xenophobia, and hate crimes against Asians and those of Asian descent have been reported in many countries. Research of He et al. (2020), which was done on a sample included 1,904 people of Chinese origin living in 70 different countries, showed that 25.11% of the participants reported having experienced discrimination without any reason identified. Africans located in Guangzhou, in southern China’ Guangdong province, suffered from acts of hostility and discrimination on the grounds that they could be the cause of a second wave of the disease (The Guardian, 2020). In India, in late March 2020, Islamophobic hashtags such as “#CoronaJihad” were shared, with Muslims blamed for spreading the virus (Perrigo, 2020). In America, blacks, non-Hispanics, and Asians reported more perceived discrimination than other racial/ethnic groups, and that this perception was highly associated with increased mental distress (Liu et al., 2020). Social media-based analyses reported an approximate 10-fold increase in the use of hateful/offensive language (Budhwani and Sun, 2020; Croucher et al., 2020; Stechemesser et al., 2020). The discriminatory discourse of certain political leaders (Human Rights Watch, 2020) has been interpreted by some as a return to a preexisting age of discrimination, especially targeting minority groups (Kim, 2020). Guterres (2020a), the United Nations Secretary-General, warned that the COVID-19 pandemic was fast becoming “a human rights crisis,” adding that “hate speech, stigma, and xenophobia continue to rise as a result of COVID-19'' (Guterres, 2020b).

Discrimination-based exclusion is commonplace in everyday life (e.g., in schools, at work, or at home) and is associated with harmful effects on both physical and mental health (Jetten et al., 2018; Haslam et al., 2019). According to Schmitt et al. (2014), perceived discrimination is negatively associated with psychological well-being (especially for members of disadvantaged groups). It is not only a violation of human rights, but also sabotages efforts to prevent the spread of the disease (Mak et al., 2006). Negative prejudice and discrimination toward certain groups can result in “positive illusion” (Busza, 2001). Thus, while individuals exclude and avoid members of a certain group, they keep in touch with members of other groups without hesitation. This in turn leads to a more rapid spread of the virus and the resultant health implications affecting human life. In extraordinary conditions such as a pandemic, it becomes necessary to fight not only the virus but also acts of discrimination. Therefore, it is vital that research and mitigation work continues in the area of infectious disease-related discrimination.

In order to deal with a problem, it is first necessary to understand its causes and the motivations behind it. Therefore, in the fight against discrimination related to COVID-19, it is important to understand the discrimination process, concepts related to this tendency, and the reasons behind such discriminatory behaviors during a pandemic. The current review aims to provide a framework in order to better understand the motives that drive such discrimination during a pandemic like COVID-19. To this end, the review first defines discrimination in detail, plus the related concepts and the main social psychological theories, which explain the basic dynamics and motivation underpinning the discrimination. Then, pandemic-related discrimination in light of past experiences is discussed and explanations put forward for the theoretical perspectives and inferences specific to COVID-19. Finally, recommendations are made in order to prevent and combat discrimination related to infectious diseases.

## Discrimination and Underlying Motivation: Perspectives of Social Psychological Theories

Discrimination is action or behavior that is directed toward members of certain groups, and is used to refer to a person or persons behaving differently (most commonly, unfairly, and humiliatingly) toward others based solely on their membership of a specific social group (Whitley and Kite, 2009). Discrimination can exhibit itself in several ways, be that verbal or nonverbal, and also in various contexts. Exclusion, racist nickname calling, threats, hostile messages, cyberbullying, obscene gestures, or physical attack are some of the more commonplace acts of discrimination. Regardless of how it manifests, discrimination leads those targeted to feel isolated, rejected, and ignored, and to experience penalty, harassment, scapegoating, and even various forms of violence.

There are certain basic concepts that are closely related to discrimination, which are “prejudice,” and “stereotype.” As these three concepts are somewhat intertwined, they are often used side by side. Generally, their relation could be summarized as labeling stereotypes as cognitive, prejudices as affective, and discrimination as the behavioral component of reactions based on the process of social categorization (Eagly and Chaiken, 1998).

“Prejudice” can be described as a generalized attitude about the features of a social group and its members. Lippman (1922) defined stereotypes as pictures in our heads that describe the features of the groups and their members. Stereotypes are generally defined by social psychologists as incorrectly biased, rigid, oversimplified, and incorrect generalizations of groups (Stroebe and Insko, 1989).

While it is clear that prejudice lies at the root of discrimination, the relationship is not always that predictable and not always linear (Whitley and Kite, 2009). Although prejudices are often based on an accumulation of experience, they can sometimes occur instantaneously based on an agenda (e.g., changes that are personal, social, economic, medical, or historical) and in these circumstances, it turns automatically to discrimination. The most striking of these relate to unexpected or extraordinary situations such as natural disasters and epidemics.

Social psychology was established as a discipline in 1908 in order to combine the micro-psychological and macro-sociological perspectives, and is considered as the beginning of an innovative framework used to examine issues faced by individuals as members of social groups (Bar-Tal, 2006). However, in the 1920s, the tendency to focus on individual-level behavior instead of collective behavior began to emerge and an academic war of wits ensued between the micro and macro perspectives in social psychology. This has also manifested itself in research studies in the area of prejudice. Within this discipline, prejudice has been traditionally characterized as an individual-level quality – “as an unfair negative attitude toward a social group or a person perceived to be a member of that group” (Dovidio and Gaertner, 2006, p 385). Although many social psychologists adopted the macro-societal context in the 1930s to early 1950s, the micro-individualistic orientation dominated throughout the 1960s and 1970s in social psychology (Bar-Tal, 2006). This reductionist tendency, led by American social psychologists, has subsequently received considerable criticism from European social psychologists. During the 1980s, powerful European theories such as “social identity” and “social categorization” (e.g., Tajfel, 1970, 1982; Turner et al., 1987), and “social representation” (Moscovici, 1984) stimulated significant repercussions. Using broader levels of analysis to include groups and societies with the perspectives of different disciplines is a complementary, rather than a competitive means to understanding the phenomenon through bridging knowledge across disciplines (Dovidio and Gaertner, 2006). A bridge must first be built between the individual and societal levels by recognizing that societies are made up of and shaped by individuals, and that individuals are social beings affected by their social environment. Also, there is a reciprocal influence between the person and the society. It is important to acknowledge, therefore, that individual-level and societal-level explanations are not mutually exclusive (Figueiredo et al., 2014).

Developing a more robust social psychology that can address ongoing social problems is still urgently required (Bar-Tal, 2006, p 345). European social psychologists have proposed a more “social” psychology with an interactionist metatheory that classifies levels of explanation, and emphasized that it should be sufficiently comprehensive to explain prejudice and discrimination through integrating the intrapersonal, interpersonal, intergroup, and ideological levels of analysis (Doise, 1986). The intrapersonal level theories deal with the way people evaluate and perceive the social world as an individual, independent of the social context. Theories at the interpersonal level address how these affective and cognitive-based evaluations regulate interpersonal relations in dual relationships or small groups. The intergroup level of analysis focuses on the cognitive-emotional and behavioral tendencies acted out as group members. Finally, the ideological level theories’ emphasis is on an individual’s way of constructing belief systems and social representations to legitimize, preserve, or challenge their status within the social structure (Brauer and Bourhis, 2006). Doise (1986) suggested that social issues should be examined using all of these four complementary levels of analysis rather than being limited to any one of them. Based on this complementary approach, in this review, first, some of the basic intra-personal and individual-level theories are addressed. Then, the more “societal” intergroup-level theories, which are considered a suitable aid for understanding the discrimination experienced during epidemics, are addressed in detail within a systematic review. Finally, the ideological-level theories are discussed.

### Intra-personal Level Theories

#### Psychoanalytic Theory

From the perspective of Psychoanalytic Theory, people tend to behave aggressively toward minority groups as a result of social (e.g., wars and famine) and individual frustrations. This can be considered a kind of “displacement of aggression.” This perspective argues that there is a motivational and adaptive dimension underpinning prejudice, and that people increase their own self-esteem through acts of prejudice, and that discriminatory behavior has an adaptive ego-defensive function (Whitley and Kite, 2009). “Scapegoat Theory” of Allport (1954) and “Ideological Theory” of Glick (2002), which took this approach to a more intergroup level, will be addressed later in Section Individual Level Theories on intergroup theories.

Additionally, according to the psychodynamic approach, as anxiety increases, others begin to be labeled as being all “bad.” This is also influenced by anxiety re-invoked from early childhood experiences (Joffe, 1999). Splitting, a deep-seated mental process, is employed as a means to coping with this anxiety. This is an unconscious defense mechanism that emerges in early childhood to keep the “bad” away from the “good” by associating good experiences with oneself, while projecting the bad outward to others. This defense mechanism comes to the surface when faced with anxiety-provoking situations such as a pandemic. Joffe (1999, p 99) asserted that the social representational framework could be complementary to connecting the sociocultural and psychodynamic explanations as responses to crises. Framework of Joffe (1996, 1999, 2003), as a psychodynamic extension of the social representation theory, posited that individuals faced with potential danger operate from a position of anxiety that motivates them to represent dangers in a specific way; linking threats to “others,” which is mainly based on the unconscious responses to anxiety. Both the self-protecting needs and the drive to externalize anxiety are grounded on a sociocultural basis. This “hybrid” model highlights the effects of the cultural context, and especially Western culture’s handling of the “individual” in terms of behavior (Joffe, 1999).

#### Evolutionary Perspective

Explaining discrimination based on the evolutionary perspective has only begun in recent decades (Whitley and Kite, 2010). According to the evolutionary perspective, discrimination is almost inevitable and therefore difficult to change as its roots lie in hunter-gatherer tribal ethos, which continues universally due to its evolutionary success (Levy and Hughes, 2009). In this framework, disease-related discrimination is an adaptive strategy and an outcome of evolved functional psychological processes that support the transmission of genes to future generations (Buss and Kenrick, 1998; Faulkner et al., 2004). These natural processes motivate people to seek to avoid contact with those suspected to be carriers of a transmittable disease. Discrimination stems from a person’s desire to guard themselves and their group against potential harm in order to enhance their reproductive fitness and, therefore, their ability to survive (Kite and Whitley, 2016, p 488). Xenophobic responses tend to increase with increased perceptions of risk in contracting a disease (Green et al., 2010). Therefore, according to the evolutionary perspective, the cause of discrimination directed toward members of a specific group is based not only according to their group membership, but also in response to a real and/or perceived threat toward their individual welfare.

Additionally, disgust is one of the basic variables related to discrimination, which the evolutionary approach emphasizes (Haidt et al., 1994), and is an adaptive emotion that serves our survival needs (Haidt et al., 1994; Kiss et al., 2020). Interpersonal disgust leads to feelings of superiority over members of the outgroup. It results in avoidance and exclusion of individuals exhibiting symptoms of an infectious disease, or are perceived as having some quality that disgusts us (Rozin et al., 2008). According to the “social contamination” hypothesis, others are perceived not only as a threat to our survival, but also as the carriers of pollution or disease, and thereby considered a threat to the integrity and purity of the ingroup (Taylor, 2007). Several studies have been conducted on the topic of disgust as a pathogen avoidance mechanism from this theoretical perspective (e.g., Curtis, 2013; Tybur et al., 2013; Lai et al., 2014). Disgust sensitivity, as an individual difference variable (Haidt et al., 1994), positively correlates with political conservatism (Inbar et al., 2012), sexual prejudice (e.g., Dasgupta et al., 2009; Herek, 2009; Kiss et al., 2020), and negatively correlates with openness (Druschel and Sherman, 1999), and all are similarly associated with prejudice and discrimination.

#### Terror Management Theory

Terror Management Theory (TMT; Greenberg et al., 1986) is a perspective on social motivation anchored in evolutionary theory (Buss, 1997; Greenberg and Arndt, 2012), which asserts that mortality salience increases the potential for experiencing existential anxiety. According to TMT, culture and religion can help some to feel a sense of control over “uncontrollable” events and thereby avoid the “unavoidable.” Mainly, they help us to cope with the reality of our being mortal. Having strong cultural worldviews, and high levels of self-esteem is seen as a way of protecting us from death-related threats (Kite and Whitley, 2016).

The motivation to support and defend the belief and value systems plays an important role in the development of prejudices (Greenberg et al., 1997). Culture has a buffer effect against the terror of mortality, and this motivates people to defend, favor, and strengthen their cultural values and worldviews during events that heighten awareness of our own mortality. “Mortality salience” provokes people to reinforce and defend both their worldview faiths and also their own self-esteem. But it also leads people to distance themselves from reminders of their own mortality. Reminders of mortality increase negative attitudes and responses toward others with different worldviews and terror management efforts increase prejudice, especially when the outgroup symbolizes a threat to people’s worldview and self-esteem. To summarize, this theory offers a unique framework “by focusing specifically on the role of existential threat in prejudice, stereotyping, and intergroup aggression” (Greenberg et al., 2009, p 309).

#### Attribution Theory

According to this theory, one of the consequences of social categorization also relates to “attributions” (Greenberg and Arndt, 2012). Heider (1958) supposed that people are “naïve psychologists,” trying to understand their social world. “Attribution” (inferring the causes of events, and our own and others’ behaviors) is one of the main processes used by humans to achieve this. Social categorization also brings about certain biases related to attributions. The most common of these, “ultimate attribution error,” could be defined as the tendency to make attributions to derogate outgroups and favor the ingroup (Pettigrew, 1979). It consists of two separate biases; (a) “explaining their own group’s negative acts by situational factors” (i.e., attributed to “bad luck”) rather than personal characteristics, and (b) explaining outgroup members’ positive acts by situational factors (i.e., to be attributed to “good luck,” or “applied effort”) rather than their personal characteristics. This error is a collective kind of “self-serving bias” (the tendency to take credit for success and deny responsibility for failure in order to protect one’s self-esteem), and “group-serving bias” (Coleman, 2013). This is a functional way of feeling superior over others, and in favoring one’s ingroup to enhance one’s own self-esteem, as explained by Social Identity Theory (SIT). These attribution biases contribute to prejudices by “viewing favorable group differences as stable and unfavorable ones as mutable” (Fiske, 2005, p 40).

On the basis of the “just world hypothesis” (Lerner, 1980) in attribution theory, humans generally believe that the world is just, that everyone gets what they deserve, and do not suffer unjustly. This way to live in a “manageable and predictable world,” can also be turned into an attribution error, which occurs by victimizing those who suffer as somehow being responsible and guilty for their own situation (Lerner, 1980). Disadvantaged groups or victims of misfortune threaten belief in a just world, and such a threat leads us to reestablish this functional belief in biasedly attributing these troubles to one’s characteristics, prior faults, or certain weaknesses. Therefore, in thinking this way, people can feel a sense of relief by believing that the same misfortunes will not happen to them if they do not behave in a way that could leave them deserving similarly (Greenberg et al., 2009).

### Individual Level Theories

#### Authoritarian Personality Approach

Following the end of the Second World War, a search ensued to answer the question of “why some people are more inclined to violence and discrimination than others.” Authoritarian personality approach of Adorno et al. (1950) emerged as one of the foremost responses to this question. People high in authoritarianism who are “strongly prone to believe and do whatever authority figures said, including treating members of derogated groups with contempt” showed racist discrimination because it was reinforced by their authority figures” (Kite and Whitley, 2016, p 34).

Right-wing authoritarianism (RWA; Altemeyer, 1981) is a theoretical refinement of this theory. People considered low in RWA (therefore; left-wing) are portrayed as ideologically liberal and supportive of social change, more open to personal autonomy, sympathize with minorities, and oppose both nationalism and racism. Whereas, the people high in RWA hold traditional and socially conservative values and religious beliefs are seen to unquestioningly obey social authorities, and are discriminative to various outgroups (Duckitt and Sibley, 2010).

#### Social Dominance Theory

Social Dominance Theory (SDT) is a relatively recent theory that handles prejudice as an individual difference (Sidanius et al., 2004; Pratto et al., 2006). This multilevel theory, which also focuses on ideological and societal factors, highlights the influence of social dominance orientation (SDO) as an individual difference that refers of peoples’ acceptance of ideologies concerning cultural equality or inequality (Sidanius and Pratto, 1999). The hierarchical social relations and social dominance within a society maintains prejudice and discrimination and, over time, legitimizes inequality. In such a society, dominant groups become disproportionately advantaged, whereas subordinate groups become simultaneously disadvantaged. This inequality begins to exist in many areas, such as political power, economic power, wealth, healthcare, leisure, educational opportunity, and also in legal rights (Pratto et al., 2006). Individuals who prefer this hierarchy have a high social dominance and low egalitarian orientation and want their ingroup to be held as superior; overall, they basically support discrimination, and are considered as authoritarian, xenophobic, racist, nationalistic, and misogynistic (Hogg and Vaughan, 2010).

While SDO and RWA may appear similar, they each have certain differences. For example, RWA stresses compliance to ingroup authority and norms, while SDO highlights the relations between ingroups and outgroups (Kite and Whitley, 2016).

#### Schwartz’s Theory of Basic Human Values

Another significant differential in prejudice is personal values. Theory of Schwartz (1992, 2007) introduced a comprehensive model that aimed to explain the relationship between these two variables. According to this theory, values guide both our attitudes and our behaviors (Bardi and Schwartz, 2003). Generally, two value orientations are associated with prejudice; individualism, and egalitarianism (Kite and Whitley, 2016). Individualism generally underlines the significance of self-confidence, but can also lead to prejudices held against certain groups and which tends to impede upon the principles of individualism. Egalitarianism, on the other hand, highlights the priority of behaving equally and fairly to all individuals and groups, so is negatively correlated to prejudice and discrimination (Abrams, 2010; Kite and Whitley, 2016).

In addition, many personality traits are known to be closely related to being to prejudice prone, for example, low levels of agreeableness and openness (Sibley and Duckitt, 2008), and high levels of religious identification (Hall et al., 2010).

### Intergroup Level Theories

#### Scapegoating Theories

The classical frustration-aggression hypothesis (Dollard et al., 1939) provides a starting point to examine discrimination as a form of intergroup behavior. Allport (1954) took this approach to a more intergroup level and developed the “Scapegoat Theory.” According to this theory, frustration causes aggression and prejudice and, generally, people tend to select some scorned outgroups as a “scapegoat” for them to blame. Glick (2005, p 244) defined scapegoating as “an extreme form of prejudice in which an outgroup is unfairly blamed for having intentionally caused an ingroup’s misfortunes.”

In line with the criticism directed toward this theory that it cannot explain why some are selected as scapegoats, while others are not, Glick (2002) developed the “Ideological Theory.” According to Glick (2002), there are certain key determinants to a group being scapegoated. These are mostly relatively weaker groups that lack the means of self-defense, are currently seen as excluded minority groups, and have visible differences such as skin color and/or gender. Scapegoating offers a designated villain for an aggressor to blame for the deprivation and frustration caused due to social and/or economic problems (Whitley and Kite, 2009). This theory addresses the perception of group-relative deprivation. If an ideology (such as Nazism) points to a scapegoat to target blame for the deprivation of a resource, it will usually meet the need of having some positive social identity. In the absence of an apparent ideology and/or a scapegoat for the deprivation experienced by the group, they will find one.

#### Realistic Conflict Theory

This approach (Sherif, 1966) proposed that individuals do not like members of an outgroup as they are perceived to compete with their own group for certain resources (e.g., economic resources, political power, social status, welfare, etc.). According to Sherif (1966), people with shared goals that require interdependence to engage in cooperation, establish a group with perceived group goals as superordinate. However, those with mutually exclusive goals tend to compete rather than form as a group, conflict, and behave discriminatively. Duckitt (1994) criticized the Realistic Conflict Theory as only explaining the competition that occurs between groups of equal status, and added that conflict frequently emerges between unequal groups such as between majority and minority groups. According to this approach, if there is no conflict, there is no discrimination. However, according to the SIT, which will be discussed, “mere existence of social groups” is sufficient for discrimination, hence there is no need for competition (Whitley and Kite, 2010, p 330).

#### Relative Deprivation Theory

According to the Relative Deprivation Theory (Davies, 1969; Crosby, 1976; Smith et al., 2012), we tend to compare our outcomes with expectations of what we perceive we deserve. These expectations are based on the outcomes of both others’ and our own past outcomes. If we evaluate our own outcomes as being low, then we may feel that we do not deserve the relative deprivation and low distributive justice. This perceived injustice and deprivation activates hostile and discriminatory tendencies toward those perceived as having caused the deprivation to occur (Whitley and Kite, 2009). Group-relative deprivation is experienced as a result of our own perception of the group as having been deprived of certain outcomes. Therefore, if we blame a specific outgroup for our own group’s deprivation, we effectively prejudice and discriminate against that outgroup group and all its members (Vanneman and Pettigrew, 1972).

#### Social Identity Theory

Social Identity Theory (Tajfel, 1978; Tajfel and Turner, 1979) focuses on the perceptual and cognitive dimensions of group membership and feelings of belonging. This theory, which has become more comprehensive over the years, and was later strengthened into the Self-Categorization Theory (Turner et al., 1987), became known as the “social identity approach” (Abrams and Hogg, 1990). SIT has four distinct components that complement each other; “social-categorization,” “social comparison,” “self-enhancement motivation,” and “people’s beliefs about relations between groups” in order to explain intergroup behavior (Tindale et al., 2001).

Social identity is defined as “the individual’s knowledge that he/she belongs to certain social groups, together with some emotional and value significance to him/her of the group membership” (Tajfel, 1982, p 31). People feel compelled to apply social categorization in order to enact a positive self-assessment and thereby enhance their self-esteem. According to this theory, people identify with their group (ingroup) and evaluate it as being of greater value, while other groups (outgroups) are deemed to be worth less. Individuals define and evaluate themselves based on the social group they belong to. In other words, they “self-categorize” (Turner et al., 1987). This classification entails identifying themselves as a member of the ingroup, which forms their social identity. The social status of the ingroup is determined by a process of biased social comparison (“us” vs. “others”), which is accomplished through the motivation to have a positive, distinct, and enduring social identity (Jetten et al., 2020). This comparison includes a biased perception by favoring the ingroup and devaluing the other groups. This process is called “ingroup favoritism” (Abrams and Hogg, 1988). People also exaggerate the similarity of ingroup members and the similarity of outgroup members to each other. With this “accentuation effect,” differences between the two groups and the uniformity between the group members also become exaggerated. Both sharpen the perception of differences between groups (Fiske, 2005) and then, become a key decisive factor of discrimination. The difficulty of distinguishing people from other races from each other is explained by this effect (Teitelbaum and Geiselman, 1997).

According to SIT, people experience anxiety and depression in the case of a threat to their self-esteem or they look for ways to deal with it. One of these ways is to develop cognitive strategies that also result in discrimination against members of groups that are suspected to be the source of the threat, which can sometimes extend to acts of hostility or violence (Vignoles et al., 2006). SIT addresses these strategies in detail.

#### Integrated Threat Theory

The three theories discussed so far (Realistic Conflict Theory, SIT, and Relative Deprivation Theory) are closely linked, and the “Integrated Threat Theory” (Stephan and Stephan, 2000) serves as a map to understand the relation between all three. This theory is based on the assumption that fear and threat are the basis of prejudice. It was developed to describe the intergroup bases of prejudice and to define the central role of the intergroup threats and fears on the process of discrimination. According to the revised version of the theory (Stephan and Renfro, 2002; Stephan et al., 2002, 2009), there are four different types of intergroup threat that causes negative evaluations of outgroups, and these are; “realistic group threats,” “symbolic group threats,” “realistic individual threats,” and “symbolic individual threats.”

“Realistic group threats” are real or perceived threats are directed “to the very existence of the ingroup (e.g., through warfare), to the economic and political power of the ingroup and the physical or material well-being of the ingroup and its members (e.g., their health)” (Stephan and Stephan, 2000, p 25). The “symbolic group threats,” on the other hand, are directed to the worldview of the ingroup. “Realistic individual threat” covers threats of actual physical and/or material harm to a group member (such as death, threats to health, economic loss, or to their personal security). Lastly, “symbolic individual threats” relate to loss of reputation or honor such as sabotaging a person’s self-identity or self-esteem.

### Ideological Level Theories

#### Social Representations Theory

Social Representations Theory is a social psychological theory of common sense understanding (Moscovici, 1984; Joffe, 1999), which focuses on the way individuals, groups, and communities collectively make sense of social issues, ideologies, and practices. It conceptualizes how socially shared beliefs and cultural values are internalized by individuals, and then how they guide them in understanding the social world (Joffe and Staerklé, 2007). Social representations are set of values, ideals, and practices resulting from the interaction between individuals, media, and social groups (Moscovici, 1984). They make the world more understandable, manageable and less threatening by facilitating the overall communication process. They present a frame of reference and guide people to make sense of the unfamiliar and unknown. This is accomplished through anchoring or classifying the unknown into already existing categories, and thereby eliminates the threat of the unfamiliar; hence, people became able to objectify it, name it, and create a social reality.

Social representations have a mediating role in the relationship between self and others; they are based on the “us-them” categorization, an essential and relatively stable opposition that underpins social representations about social groups (Staerklé, 2015). They act also as a form of social identification (Prislin, 2010, p 581). They are prescriptive and persistent, having been established historically and connected to our collective memory and culture to work as a form of background context (Andreouli et al., 2014). This theory also helps us to understand the social processes underlying legitimacy and social order. Social representations have been figured as specific types of knowledge facilitating communication and organizing social relations (Staerklé, 2015). The “social representation” and “social order” concepts are closely intertwined. According to this theory, people look for a shared frame of reference in order to adapt to the world around them and for their interaction with others. It allows studying the “passage of knowledge from scientific thinking, *via* the mass media, to lay thinking” and focuses on the role of the media in forming a common sense at the group level (Washer and Joffe, 2006, p 4). Several studies have been published that have applied this framework in order to explore how society deals with risks such as addiction (Farrimond and Joffe, 2006), climate change (Moloney et al., 2014), infectious diseases like AIDS (Joffe, 1999; Joffe and Bettega, 2003), Ebola (Joffe and Haarhoff, 2002; Idoiaga Mondragon et al., 2017), SARS (Washer, 2004), MRSA (Washer and Joffe, 2006; Washer et al., 2008), and Avian Influenza (Joffe and Lee, 2004), among others.

#### System Justification Theory

System justification theory (SJT) focused originally on prejudice and intergroup relations, and was later expanded to explain the general human tendency (especially members of disadvantaged groups) to support and defend the social *status quo* (Jost and Banaji, 1994; Jost and van der Toorn, 2011, 2012). System justification has a palliative function that increases legitimizing the *status quo* and satisfies epistemic, existential, and relational needs, which diminish uncertainty, threat, and social conflict (Jost, 2019). Jost and Banaji (1994) suggested that the well-known motives of ego justification (self-interest) and group justification (ingroup favoritism) were insufficient to explain intergroup behavior. SJT adds a third; motives of system-justification, as in the tendency to defend and justify the systems to which an individual (or even members of disadvantaged groups) belong. Just as some defense mechanisms come into play when there is a threat to our self-esteem or to our social identity, system justification motives become apparent when a threat is perceived to the legitimacy of the system to which we belong (Blasi and Jost, 2006, p 1123). This kind of tendency attributes more positive traits to privileged members of society at the cost of seeing their ingroup more negatively referred to as “outgroup favoritism.” This is a system-justifying bias because having the potential to reinforce and make permanent inequality, especially when these attitudes are held by disadvantaged groups (Caricati and Owuamalam, 2020). In this way, stereotypes help to maintain hierarchical social arrangements (Blasi and Jost, 2006).

According to SJT, most political ideologies are located on a left-right, or liberal-conservative dimension. The liberal mind rejects social inequality, hierarchy, and discrimination; while the conservative mind resists social change, endorses social inequality, and prefers traditional values and hierarchy. Consequently, system justification is more marked among conservatives. They justify and protect the *status quo* even if it means upholding an unfavorable position for their ingroup. This irony can be evaluated as a result of the need for uncertainty reduction (life with the ongoing circumstances is better than an uncertain future) and to avoid cognitive dissonance (Hogg and Vaughan, 2010).

## Pandemic-Related Discrimination: Past Experiences and Implications From Theories to Covid-19-Related Discrimination

During the H1N1 pandemic, across Europe and Malaysia, specific groups, such as the homeless, homosexuals, and those perceived as living a promiscuous lifestyle were faced with prejudice and discrimination (Goodwin et al., 2009). Individuals stigmatized with HIV/AIDS and TB have become disadvantaged in terms of healthcare services and employment, restricted entry to many countries, and ill-treated by their neighbors and colleagues. This kind of discrimination has also been documented for SARS, syphilis, and also for genital herpes. Infectious-related stigma and discrimination are defined as being overwhelming to individuals with, or even suspected of having, the infection as the diseases themselves (Mak et al., 2006). It was reported that in Thailand, almost 10 years after the AIDS pandemic (June, 1999), those orphaned are still coerced into leaving their settlements, HIV-positive children still barred entry to schools, and some health centers continue to decline to treat people infected with HIV/AIDS. During the same period, in Cambodia, even families have been known to reject HIV-positive family members, while in Bali, they have been forced into isolation along with their whole family (Busza, 2001). Due to the high probability of spread, morbidity, and mortality, those with or suspected to carry or suffer from infectious diseases are known to be stigmatized (Malcolm et al., 1998; Lau et al., 2005). Regardless of whether or not they are infected, people are exposed to discrimination more often than usual during epidemics based upon the group to which they belong, the region or country in which they live, their race, ethnicity, or their religious beliefs. As a result, individuals who have been discriminated against have become increasingly vulnerable, and those who are infected find it harder and slower to recover (Williams et al., 2011).

It is essential to understand the motivation leading to visible increases in discrimination during pandemics, and especially COVID-19-related discrimination. In this section, inferences are made about both pandemics in general and COVID-19 in particular, and are discussed based on explanations of the aforementioned theories which specific, parallel, and complement each other according to previously published research on disease-related discrimination.

### Implications From Intra-personal Level Theories

The COVID-19 pandemic created “frustration” and “deprivation” in many areas of life due to its high level of contagiousness and its impact that brought life to a near halt with severe restrictions imposed on modern societal freedoms. From the perspective of *psychoanalytic theory*, these social and individual frustrations can be the cause of aggressive attitudes aimed at minority groups (Whitley and Kite, 2009). As previously mentioned, the classical frustration-aggression hypothesis (Dollard et al., 1939) has been the starting point for many new approaches to discrimination like Scapegoat Theories (Allport, 1954; Glick, 2002) and, accordingly, being from a minority group or one with visible differences such as skin color are seen as vulnerabilities to being considered a scapegoat (Glick, 2002). As numerous studies have shown (e.g., Devakumar et al., 2020; He et al., 2020; Liu et al., 2020; Perrigo, 2020), these explanations became significantly visible during the COVID-19 period, with high levels of economic and social deprivation experienced during the novel pandemic function as a form of frustration.

From the *evolutionary framework*, discrimination, avoidance and exclusion are evolved adaptive responses aimed at protecting individuals from the threat of diseases (Faulkner et al., 2004; Gilles et al., 2013). Discrimination does not unconsciously just emerge, but serves a very specific purpose. Also, individual differences observed in terms of “perceived vulnerability” and “aversion to germs” seem to predict prejudices against foreigners (especially minorities and immigrants; Duncan et al., 2009). A recent study with an American sample (Tabri et al., 2020) revealed that the existential threat stemming from COVID-19 elicited anxious arousal, and indirectly predicted subtle and blatant prejudice toward people from China or those perceived to be of Chinese heritage, which were perceived as a source of the threat.

We can explain infectious disease-related discrimination by human survival instincts, and the drive for preservation of personal and public health. The emergence of viral outbreaks create an existential threat at both the individual and societal level, having characteristics that are inherently unknown and dangerous due to uncontrolled rapid transmission, which generates a near-instantaneous impact on daily life. To lessen the probability of extinction, individuals and outgroups with certain qualities become stigmatized as patients and transmitters, and hence face acts of discrimination.

Infectious-disease-related discrimination becomes much more understandable based on the perspective of *TMT*. Due to its rapidly increasing death toll, the COVID-19 pandemic is likely to activate mortality salience (Courtney et al., 2020), which has also been shown to increase biases against other groups. Considering the increase in mortality, an increase in intergroup bias is also expected (Harmon-Jones et al., 1996). Becker (1975) stated that fear of death leads to hostility toward outgroups because they endanger our immortality illusions. This tendency, which is another way to tackle a dread of mortality, shows itself by despising the scapegoat as being “less than human,” who does not deserve equal rights, and is viewed by ingroup members who see themselves as more qualified and “true humans.” This is used by the ingroup as a goal to affirm control over life and death, a way of symbolically securing themselves against the ravages of disease and death (Greenberg et al., 2009). Ageism has become one of the most commonly observed types of discrimination exhibited during the COVID-19 outbreak (Ayalon et al., 2020; Brooke and Jackson, 2020; Rahman and Jahan, 2020), due in part to harsher restrictions and forced isolation imposed on older individuals due to their vulnerability to the effects of the virus. It can be said that children also experienced their fair share of ageism during the epidemic, especially after having been classified as a “non-at-risk age group” during the early stages of the pandemic. Ageism is prejudice based on age and most research conducted on this topic has focused on discrimination against older adults. This stems from gerontophobia; an irrational fear, hatred, or other hostility toward older adults. These people are viewed by some as salient reminders to their younger self of their own mortality, so they automatically apply mortality salience. The youth formulate a kind of defensive buffer by disparaging the elderly to cope with their own mortality fears. As Nelson (2016, p 347) noted, ageism “is our own prejudice against our feared future self.” Infectious-disease-related discrimination can lead to the marginalization of certain at-risk groups as a means of coping with fear. People live with a “positive illusion” and begin to evaluate those from certain “other” groups as being more “at-risk” than themselves. This illusion lets them feel that they can escape their fear, but, as they underestimate the risk of contracting the disease due to this misconception, they begin non-compliance with preventive health behaviors and precautions and thereby place themselves and others at greater risk as a result (Busza, 2001).

Based on the *Attribution Theory* (Heider, 1958), individuals’ attributions of the cause of disease determine their responses toward the real or perceived disease carriers. On this point, the perception of “controllability” seems strongly linked with stigma and discrimination (Weiner et al., 1988). The public attributes responsibility for their illness to the suspected groups, and will therefore blame them and discriminate against them with the disease’s spread labeled as “controllable by the individuals.” Mak et al. (2006) revealed that increased stigmatization and blaming of infected people and their groups can be observed in cases where a patient’s disease was attributable to their own carelessness or irresponsibility (internal attribution), rather than the disease being interpreted as uncontrollable (external attribution). To summarize, it is mainly as a result of biased internal attribution that some are discriminated against due to being somehow responsible for their differences, while others are not.

In a just world, everyone gets what they deserve, where “bad things happen to bad people” and “good things happen to good people” (Burger, 1992; Greenberg et al., 2009; Jost and van der Toorn, 2012). During the COVID-19 pandemic, many people evaluated the onset and spread of the outbreak in this way. Many opinions were put forward that the spread of the disease was caused by some people’s seemingly unusual dietary habits, and that they deserved what happened to them as a result; or that God had punished some people in this way because of their moral weaknesses, or that Mother Nature was punishing those who mistreated her.

We have stated that another outcome of social classification relates to the “attribution” process, as in perceiving the inner group and oneself as superior. “Group-serving bias” (Coleman, 2013) is also a common way to favor the ingroup and thereby enhance self-esteem. According to “Attribution-Value Model” of Crandall et al. (2001), prejudice and discrimination are the output of seeing minority groups as having opposite characteristics to the values of the majority group. Thus, those with a body condition classed as clinically obese are seen as lazy and weak-willed individuals, and those suffering from AIDS are deemed polygamous and immoral (Joffe and Staerklé, 2007). Similarly, those who contracted COVID-19 may be seen as people who “eat anything.”

### Implications From Individual Level Theories

During the COVID-19 pandemic, personal differences in terms of discrimination tendencies were seen related to certain characteristics such as *authoritarian personality*. Societal threats like pandemics bring about increases in the support of authoritarian beliefs (Green et al., 2010). National identity becomes more salient when global crises like pandemics come to the fore, and is therefore the strongest determinant of xenophobia (Brown, 2000). As previously addressed, being authoritarian and endorsing social hierarchy is one of the main predictors of prejudice and discriminatory tendencies (for details, see Pratto et al., 2006). Both *RWA* and *SDO* are founded as predictors of prejudice and intolerance (Altemeyer, 1981; Thomsen et al., 2008). Recently, Hartman et al. (2020) found that the existential threat that stemmed from the COVID-19 pandemic led to associations between RWA and nationalism, and anti-immigrant attitudes conditional on levels of perceived threat. To summarize, it could be said that the COVID-19 pandemic activated authoritarianism in society and thereby triggered discrimination (Hartman et al., 2020).

Implications from intergroup level theories.

According to Muldoon (2020), during the novel pandemic, physical distancing, self-isolation, food access, and hygienic living conditions became more inaccessible or only considered as a luxury for many, which further exacerbated their inequality and vulnerability. Muldoon (2020, p 85) summarized this by saying, “Life in 2020 will be vastly different if you are a nurse rather than an academic, a New Yorker rather than a New Zealander, or aged 80 rather 20.” *Social Identity Approach* (Tajfel, 1978; Turner et al., 1987) is functional in understanding these dynamics, with group membership a crucial factor that predicts each person’s COVID-19 pandemic experience both psychologically and structurally.

In trying to determine their own group’s response to the COVID-19 pandemic, people regularly monitor the number of infected and lives lost, according to various media channels, that is, and then make a social comparison (Jetten et al., 2020). Thus, they evaluate their position according to their country, city, or region, and then relax or tighten their adherence to the established rules or guidelines. After this comparison, it is possible to apply temporary relief by applying a downward comparison; in other words, making the choice to compare against worse-off groups (for details, see Festinger, 1954). This social comparison includes ingroup favoritism as previously mentioned. Ingroup favoritism and accentuation effect can significantly trigger discrimination (Fiske, 2005). During the COVID-19 pandemic, it could be said that, as in previous examples of widespread infectious diseases, that these biases lead people to see their own group as being more superior, while seeing other groups as less worthy than their own (Green et al., 2010; Joffe et al., 2011; Assche et al., 2020). In this way, individuals who discriminate against certain groups aim to strengthen their own social identity and self-esteem, which can be said to be a means of coping with the anxiety of having contracted or been potentially exposed to the disease.

As stated by Cogan and Herek (1998), pandemics prepare the ground for acts of discrimination if the cause is regarded as being attributable to a specific individual or a certain group; if it is thought to be terminal or degenerative; if it is considered to be contagious or detrimental to others; or, if it is considered highly visible. Research has shown that during pandemics, outgroup members are mostly blamed for carrying and spreading the disease, and that the responsibility is therefore squarely attributed to them. As a result, accusatory and discriminatory behaviors increase, and such discrimination can be reflected in the sanctions applied to those who do not comply with the pandemic measures. In a recent study, Assche et al. (2020) found that individuals who strongly advocated for COVID-19 related retributive measures supported their application more for outgroups than for members of their own ingroup.

According to Vignoles et al. (2006, p 310–311), there are five more motivations to social group identification besides maintaining and enhancing self-esteem; a need for “efficacy” (to maintain or enhance feelings of competence and control), a need to “belong” (to maintain or enhance feelings of closeness to, or acceptance by, other people), a need for “distinctiveness” (motivation to maintain the sense of differentiation from others), a need for “continuity” (motivation to maintain a sense of continuity across time and situation), and a need for “meaning” (to find significance in and purpose for one’s own behaviors and existence). According to this approach (Vignoles et al., 2006), the more the individual’s social identity satisfies these needs, the more it becomes an important part of their self-concept and thus, their identification with the group will increase. They also proposed that when people face a threat, they take into account how far the situation is likely to prevent them from satisfying each of these six needs. As their level of deprivation increases, they focus more on their social identity and begin to differentiate more between “us” and “them.” According to the *Relative Deprivation Theory*, blaming an outgroup for the ingroup’s deprivation causes anger, resentment, and discrimination (Vanneman and Pettigrew, 1972; Smith et al., 2012).

As developed to explain the central role of the intergroup threats on prejudice, the *Integrated Threat Theory* (Stephan and Stephan, 2000) has emerged as a theory that has grounded disease-related discrimination research in recent years (e.g., Navarrete and Fessler, 2006; Schaller, 2006; Green et al., 2010, 2020; Croucher et al., 2020). A considerable amount of research has shown that the perception of the intergroup threat is one of the main antecedents to discrimination (Stephan et al., 2009; Green et al., 2016; Visintin et al., 2020).

The COVID-19 pandemic is openly a typical “realistic threat,” which threatens the welfare of groups worldwide. However, it also poses a symbolic threat because of the social distancing measures, which have led to the weakening of the sense of community and social identity. Research of Kachanoff et al. (2020) revealed that both realistic and symbolic threats of COVID-19 predict higher levels of distress and lower perceptions of well-being.

Circumstances that threaten the welfare of the group, in turn, can result in increased identification with the group. Especially, real or perceived threats to the group’s survival (of which a pandemic is an example) can also lead to the same result. Prejudices and discrimination against foreigners (especially immigrants) rises in cases of increased national identity (Kite and Whitley, 2016). This can be extreme in the case of a national identity based on an ethnicity rather than the civic view of nationality, or in the case of the combination of “group narcissism” (a belief in the superiority of one’s own country and its culture over all others, coupled with denial of its negative aspects) and “national identity,” which can form an elevated level of prejudice against outsiders due to a perceived threat to their country’s welfare (Kite and Whitley, 2016, p 11).

During events such as pandemics, the costs and benefits of interacting with the ingroup vs. outgroups can also determine the attitudes exhibited toward each group. Interaction with ingroup members will be perceived as inherently less risky in terms of disease transmission than would interaction with members of outgroups. On the other hand, interaction with ingroup members has some obvious vital benefits like the provision of aid to each other should the disease be contracted. Health-related threats have also other adaptive features that strengthen the ingroup ties and the sense of unity. Besides, infectious disease-related prejudices and discriminative acts are associated with certain personal variables, especially a perceived vulnerability to contracting an infection (Green et al., 2010). Navarrete and Fessler (2006) found that ethnocentric attitudes increase as an outcome of perceived disease vulnerability. Faulkner et al. (2004) revealed that feelings of vulnerability to infection motivate xenophobic attitudes and negative reactions to foreigners.

### Implications From Ideological Level Theories

The *SJT* claims that discrimination is based on satisfying the needs of “self-esteem” and “need for control.” With regards to pandemics, nationalism (Nelson et al., 1997) and the justification of hierarchy (Landau et al., 2004; Hirschberger, 2006), which are significantly related to discrimination, become more widespread and strengthened. These “system-justifying” biases having the potential to reinforce inequality and make them permanent, especially when such attitudes are held by disadvantaged groups (Caricati and Owuamalam, 2020).

According to Green et al. (2010, p 301), the *Social Representational Approach* complements the evolutionary theory by helping to understand the collective sharing and cultural transmission of fears. Shared beliefs on emerging epidemics constitute collective coping strategies as a means to dealing with a threat. Explanations of the social representations theory are specifically important considering the fueling role of the media in increasing discrimination (Budhwani and Sun, 2020; Croucher et al., 2020; Stechemesser et al., 2020) in the COVID-19 process. According to this approach, crises like epidemics greatly affect the representations of outgroups and the need to distinguish between “us” and “other” intensifies. People tend to dissociate themselves from epidemics and link them with others. Certain groups categorized as “other” are blamed for the disease and thereby became dehumanized as being represented as non-human, negatively valued creatures as vermin, bacteria, or maggots (Joffe, 1999, p 22). Washer and Joffe (2006) asserted that representations of emerging infectious disease are rooted in the externalizing of the threat by linking the disease with the “other” and through blaming members of the outgroup. According to Joffe (1999), control rather than indulgence is the core norm in Western society. In these cultures, “the other” arouses fear, and is represented as being antithetic to highly valued features, such as self-control, self-denial, and self-discipline (Joffe and Staerklé, 2007). People with substance abuse disorders, homosexuals, and those with contagious diseases are stigmatized for not having these values. Representations of health and disease are based on cultural background and are constructed through communication, social interaction, and also daily experiences (Jovchelovitch and Gervais, 1999, p 237). When the emergence of a new threat is announced through the media, inferences about this information are made and its social representation starts to be formed. This representation serves not only to understanding the new phenomenon, but also in finding a specific collective for which to blame (e.g., nations, ethnic groups, professions, or social categories such as those with substance abuse disorders) for this new risk (Mayor et al., 2013). Unfamiliar objects/events activate feelings of threat and people choose to “accommodate” it into an already existing approach. Emerging new infectious diseases trigger the need to distance from outgroups in order to preserve the perceived “purity” of the ingroup (Green et al., 2010). There are several examples throughout human history of this “symbolic othering” (Joffe, 1999) process, which could form a guiding concept to understanding discrimination related to COVID-19.

Both media-based news sources and casual informational resources can be the cause of fear and panic in many people, and this emotional tension creates the potential for discrimination toward particular groups. An “infodemic” refers to information supposedly based on fact that lacks validity and is spread *via* social media as discriminatory viewpoints such as has been seen extensively in the case of COVID-19. From the emergence of the novel pandemic, anti-Chinese tendencies were triggered globally based on conspiracy theories, condescending posts about cultural norms and the dietary habits of the Chinese people (Dubey et al., 2020). Blaming a group for an issue is anchored in already existing representations, so old representations continue their influence and power in new similar ones (Moscovici, 1961). Novel diseases activate the perception of threat and individuals accommodate it into an already existing representation (Tanner, 1997). Attributions of poor hygiene and dietary habits can be employed to anchor a novel pandemic within existing representations (e.g., derogatory representations of low status outgroups; Gilles et al., 2013).

## Discussion

According to Barrett and Brown (2008), the “stigma epidemic” could spread faster and farther than the pandemic itself, and as a result, cause numerous medical, social, political, and economic problems. Pandemic-related discrimination is not only a violation of human rights but also delays and damages the efforts exhibited to prevent the spread of the virus. Besides the obvious harmful consequences for the individuals targeted, it also influences the spread of the virus by negatively affecting the public’s attitude toward prevention and restriction, health service procurement, and in the establishment and application of health-related policies. Therefore, this is a crucial issue that requires and deserves significant emphasis.

The development of discrimination and inequalities are related to many variables including cultural, educational, political, religion, personal, economical, and environmental issues. The social sciences, especially Social Psychology, Sociology, Clinical Psychology, and Health Psychology mainly conduct research studies and develop comprehensive theories on prejudice and discrimination. However, this multivariability requires a more multidimensional perspective, and especially so in the case of pandemics. The wealth of conceptual and theoretical accumulation of social psychology can provide a guide to understanding the individual, group, and state responses related to COVID-19, and to designing and implementing anti-discrimination programs that endeavor to prevent and reduce instances of disease-related discrimination (Smith and Gibson, 2020). Understanding the motivations that underlie this specific form of discrimination is critical, not only for the design of anti-discrimination programs, but also for the protection of public health.

As this review summarizes, there are many factors involved that motivate people to discriminate, with some acts serving specific functional purposes. However, this does not mean that we must tolerate and accept such behaviors, nor should we evaluate them as right or excusable (Kite and Whitley, 2016, p 40). Raising people’s awareness and tackling discrimination can take many years, and in some cases is never eliminated (Ainlay et al., 1986). As previously mentioned, prejudices have a very persistent nature. It is well-known that it is vital to diagnose and take precautions early on in order to effectively manage difficult-to-treat diseases. The same approach is necessary for dealing with discrimination.

New innovative interventions need to be designed in order to cope not only with new pandemics, now and in the future, but also infectious disease-related discrimination. It is vital to eliminate discrimination on a global scale by adapting the suggestions of the research together with the experience of past pandemics and from COVID-19, taking into account their unique features in order to best apply the knowledge that exists in the published research. Managing crises and preventing panic, fear, and feelings of desperation to appropriate levels will help to reduce prejudice and discrimination both during and following an epidemic.

As Parker and Aggleton (2003, p 17) noted, understanding these experiences and their outcomes can guide us to develop better measures for combating and reducing these negative effects. It is important to understand how social categorization and related phenomena are used by individuals and groups to create inequalities and injustices. Research on the dynamics of discrimination and stigma can help with the development of programs aimed to combat discrimination (Mak et al., 2006).

It is crucial, therefore, to ensure that health coverage is made fair for all, to pursue policies that are free from hate speech and discrimination, and to protect vulnerable groups that are often scapegoated during health-related crises. In the case of COVID-19, while we should not forget that the pandemic will come to an end at some point, there is, however, no vaccine for discrimination, and its traces remain, only to resurface time and time again.

## Recommendations for Policymakers and Stakeholders

COVID-19 has been evaluated as the largest global crisis since World War II. The disease presents a form of collective trauma that is caused by threat to health, life, and safety, and is common to all people around the world (Muldoon, 2020). In this section, based on all these explanations and lessons learned, some suggestions, which are based on the social psychological perspective, are put forward for the attention of those responsible for managing the process, for both today and for our collective future.

First of all, it is the world’s governments who are tasked with managing the course and effects of a pandemic crisis. In a study that compared public stigma toward three types of infectious disease (HIV/AIDS, SARS, and Tuberculosis) in Hong Kong, Mak et al. (2006) revealed significant relationships were established between stigma and public attitudes toward government policies. Experiences during the COVID-19 pandemic have shown that in the earliest stages of an epidemic, governmental transparency must be clearly established. Misinformation, suspicion, and uncertainty all go toward increasing discrimination, which can in turn cause panic among the public.

*From the intra-individual level perspective*, by raising the general publics’ awareness of the potential sources of anxiety and frustration they may be facing, discrimination augmented by the defense mechanisms of displacement and projection, the triggered sense of terror created by mortality salience, and the adaptive avoidance and disgust toward perceived disease carriers may be prevented to a certain extent.

*From the individual level perspective*, it has been seen that authoritarian beliefs receive greater levels of support during pandemic periods (Green et al., 2010). Both RWA and SDO are known predictors of prejudice (Thomsen et al., 2008). Existential threat related to COVID-19 has been found to be associated with authoritarianism, RWA, nationalism, and anti-immigrant attitudes (Hartman et al., 2020). The outward attitudes of politicians and community leaders are therefore of vital importance, especially, where authoritarian personality types are considered. Leaders can drive citizens toward discriminatory behaviors through the formation and application of poorly judged or narrow focused policies. It is therefore vital to ensure that sensitivity is applied with regards to discriminatory behaviors during such extraordinary circumstances as a pandemic, to try to raise awareness, and to focus efforts on prevention measures as the priority. For this purpose, politicians should actively seek counsel from social scientists, educators, and media actors. In all countries, managing an efficient, reliable, and persuasive health communication, the cooperation of healthcare professionals and the media is key to the delivery of accurate information critical to the prevention of an “infodemic” (Shimizu, 2020). A multidirectional psychosocial preparedness specific for potential future pandemics is therefore required (Dubey et al., 2020).

*From the intergroup level framework*, it has been seen that the inequalities that existed during this process have become more evident, and that lower-status group members have become even more vulnerable as a result. Also, it is necessary to consider this issue more deeply in terms of its effect on minorities (e.g., Green, 2007; Fasel et al., 2013; Pareek et al., 2020). Policies should therefore be developed in order not to increase or exacerbate this even further, and to make the “group” emphasis on the axis of “humanity” identity as a means to minimizing the damage caused by discrimination. It is only possible to win this war by seeing the virus itself as a threat for all humanity, and by evaluating humanity as one singular entity through a “superordinate level of categorization” (see Tajfel and Turner, 1979). As previously mentioned, intergroup threats bolster commitment to our ingroup (e.g., Castano et al., 2002; Greenaway, 2020). Leaders also reinforce this and overemphasize being “us” in their public speeches and policies. This is very functional from this point of view as a means to dealing with the uncertainty and fears specific to a pandemic. However, this emphasis should be inclusive, not exclusive, and not trigger discrimination as a result. Intergroup threat also defines “who is inside and who is outside,” while strengthening social identity and increasing ingroup solidarity. Therefore, political and societal leaders must be made aware that representing COVID-19 as an intergroup threat has the potential for certain potentially serious negative outcomes as well. In terms of Realistic Threat Theory, there is no doubt that realistic and symbolic threat perception strengthens during the pandemic process, and as a result triggers discrimination against immigrants and minority groups in general (Schlueter and Scheepers, 2010). Therefore, it is necessary to make concerted efforts to change this perception in order to fight pandemic-related discrimination. Research has shown that intergroup contact can reduce the perceived threat, and that this reduction brings about a decrease in negative prejudices (Stephan and Stephan, 2000; Pettigrew et al., 2007; Harwood et al., 2013; Green et al., 2020). Research of Mandalaywala et al. (2020) with an American sample on anti-Asian prejudice related to COVID-19 showed that intergroup contact was significantly associated with lower levels of discrimination regardless of the actual or perceived threat. However, contact is not always sufficiently constructive to provide efficient context to elicit positive attitudinal change, and may even lead to opposing results in the case of unexpected bad outcomes (Stangor et al., 1996). Additionally, another problem often seen is overgeneralization. It is common to see individuals in contact as an exception, to evaluate them as a “subtype” and then not to generalize the positive attitude change to aim at the whole group (Stangor, 2009). There are different qualities of contact (contact quantity, contact quality, cross-group friendships, face-to-face, virtual and parasocial, extended, and imagined) that each have varied effects on intergroup conflicts (Harwood et al., 2013; Visintin et al., 2020). It would therefore be of significant importance to take benefit from the findings of recent extensive research on this subject (e.g., Green et al., 2016, 2020; Kende et al., 2017; Visintin et al., 2020) in order to determine which type of contact is more appropriate to this process, and how the conditions should be determined. Intergroup contact is not some form of magic that will end intergroup conflicts and discrimination (Al Ramiah and Hewstone, 2013), but it has been proven that contact does not usually further antagonize intergroup relations, and generally develops them in a positive manner (Pettigrew and Tropp, 2006).

*From the ideological level perspective*, it has been seen that nationalism (Nelson et al., 1997) and the justification of hierarchy (Landau et al., 2004; Hirschberger, 2006) become more widespread and strengthened during the pandemics. The “us” and “others” divisions increase sentiments of nationalism, and nationalism strengthens prejudices and discrimination, which places international relations in jeopardy among other outcomes (Assche et al., 2020). In extreme situations like a global pandemic, people become more and more attached to their social identity (Dovidio et al., 2020). It is seen that political leaders frequently emphasize social and national identity during this process. However, emphasis on international solidarity, the sense of unity and “we-ness” would help to reduce tension and acts of discrimination, and help in the united fight against the one common enemy, the virus. Effective management of the COVID-19 crisis requires global leaders who aim to create international unity and care for the interests of humanity as a whole, and not just focusing on national or party-based interests, despite all the material and moral difficulties (Jetten et al., 2020). In not doing so, international tensions and negative social representations will rise and continue to so, and they will survive for many years to come. As difficulties and uncertainties in controlling a viral epidemic or pandemic increase, it has to be realized that national leaders’ should leave aside partisan leadership and highlight the strengths of the union of the country that they govern, as well as for humanity as a whole (Haslam, 2020). When political leaders take steps based on party lines, polarizations will naturally arise and tensions fueled that may trigger intergroup hostilities and discrimination, and will also hamper any successes in the fight against an epidemic. During this process, their own political status or party line can no longer be the primary focus, but the national welfare of all citizens in their care (Crimston and Selvanathan, 2020).

Measures taken by policymakers need to be introduced much faster, and acted upon much quicker in order for their effect to trigger any noticeable and beneficial change. However, it would be more rational to attribute the responsibility for such measures to be taken not only to a specific group or leader, but through the cooperation of all leaders across the political spectrum, as well as international health organizations, the global media, non-governmental organizations, and opinion leaders (Abdelhafiz and Alorabi, 2020).

At the final word, being aware of our tendencies is a good start to any fight. It is therefore of significant importance to understand prejudice and discrimination, and to understand how all the relevant processes work in high-threat conditions such as a pandemic, and to develop and implement appropriate, up-to-date, and forward-looking measures as required. As human beings, we are all in this together, and by banding together in working toward a “collective cure,” we could help society and our species to overcome this trauma (Muldoon, 2020).

## Author Contributions

The author confirms being the sole contributor of this work and has approved it for publication.

### Conflict of Interest

The author declares that the research was conducted in the absence of any commercial or financial relationships that could be construed as a potential conflict of interest.
